# Antibiofilm Strategies in Neonatal and Pediatric Infections

**DOI:** 10.3390/antibiotics13060509

**Published:** 2024-05-30

**Authors:** Chrysoula Kosmeri, Vasileios Giapros, Anastasios Serbis, Foteini Balomenou, Maria Baltogianni

**Affiliations:** 1 Department of Pediatrics, University Hospital of Ioannina, 45500 Ioannina, Greece; chrisa.kosmeri@gmail.com (C.K.); tasos_serbis@yahoo.com (A.S.); 2 Neonatal Intensive Care Unit, School of Medicine, University of Ioannina, 45110 Ioannina, Greece; faybal18@yahoo.com (F.B.); mbalt@doctors.org.uk (M.B.)

**Keywords:** biofilms, infections, neonates, children, treatment strategies

## Abstract

Biofilm-related infections pose significant challenges in neonatal and pediatric care, contributing to increased morbidity and mortality rates. These complex microbial communities, comprising bacteria and fungi, exhibit resilience against antibiotics and host immune responses. Bacterial species such as *Enterococcus faecalis*, *Pseudomonas aeruginosa*, *Staphylococcus aureus*, and *Staphylococcus epidermidis* commonly form biofilms on medical devices, exacerbating infection risks. Neonates and children, particularly those in intensive care units, are highly susceptible to biofilm-associated infections due to the prolonged use of invasive devices, such as central lines and endotracheal tubes. Enteral feeding tubes, crucial for neonatal nutritional support, also serve as potential sites for biofilm formation, contributing to recurrent microbial contamination. Moreover, *Candida* species, including *Candida pelliculosa*, present emerging challenges in neonatal care, with multi-drug resistant strains posing treatment complexities. Current antimicrobial therapies, while important in managing infections, often fall short in eradicating biofilms, necessitating alternative strategies. The aim of this review is to summarize current knowledge regarding antibiofilm strategies in neonates and in children. Novel approaches focusing on biofilm inhibition and dispersal show promise, including surface modifications, matrix-degrading enzymes, and quorum-sensing inhibitors. Prudent use of medical devices and exploration of innovative antibiofilm therapies are imperative in mitigating neonatal and pediatric biofilm infections.

## 1. Introduction

Biofilms represent communities of microorganisms comprising bacteria, fungi, and strains, exhibiting a sophisticated ecological phenomenon within medical environments. These microbial clusters adhere to surfaces through an extracellular polymeric substance which consists of a complex mixture of proteins, polysaccharides, and extracellular DNA [1]. Particularly noteworthy is the propensity of medical devices to serve as the breeding grounds for biofilm formation, offering an optimal foundation for the colonization of bacteria and fungi [2].

Neonates and children, particularly those under intensive care, confront heightened susceptibility to infections linked with biofilms. This vulnerability stems from their underdeveloped immune systems and frequent exposure to invasive medical procedures and devices [3]. Furthermore, the protracted duration of hospital stays, often necessary in neonatal and pediatric intensive care, further increases the risk of biofilm-forming pathogens thriving within healthcare settings.

Central venous catheters, peripheral central lines, endotracheal tubes, urine catheters, and enteral feeding tubes are among the common locations of biofilm-associated illnesses in neonatal and pediatric populations. Additionally, umbilical arterial and vein catheters are also possible sites of biofilm formation in neonates. Invasive devices may facilitate the acquisition and transmission of organisms which are able to attach and form biofilms on non-living surfaces. These biofilms usually start from the medical device’s surface or insertion point, either via hematogenous dissemination, local colonization, cross-contamination, or infusion of contaminated items such enteral feeds. Particularly, parenteral nutrition and enteral feeds act as the breeding ground for bacteria due to their high glucose content.

Biofilms of bacteria are remarkably resistant to antibiotic therapy and host immune cells [4] due to their protective matrix, which shields them from pH changes, food shortages, and mechanical stresses [5,6,7]. As a result, biofilms play a significant role in the development of resistant chronic illnesses. For bacterial infections to spread and survive within the host, complete biofilm production is essential [7]. An estimated 50% of nosocomial infections are connected to indwelling medical devices, and biofilms have been implicated in a considerable proportion of bacterial infections in people [8]. As a result, biofilm infections provide considerable challenges and raise the rates of morbidity and mortality.

Preventative measures against biofilm infections in children and neonates emphasize strict adherence to infection control protocols, prudent use of antimicrobial agents, regular monitoring, and maintenance of medical devices, along with minimizing the duration of invasive procedures and device usage whenever feasible.

The aim of this narrative review is to summarize the well-established and novel knowledge regarding the prevention and treatment of biofilm infections in neonates and in children. The PubMed and Google Scholar databases were searched for ways to combat biofilm infections in neonates and children. The following terms were used: biofilm, neonatal infection, pediatric infection, intensive care unit, treatment, prevention, combating biofilm infections. Peer-reviewed studies published up to April 2024 were included, particularly observational studies and systematic reviews, when available. Additionally, the reference lists of the retrieved articles were examined to identify any relevant studies that might have been missed in the initial search. In absence of available studies in children and neonates, studies conducted in the adult population were also included in the discussion of this review.

## 2. Biofilm Formation

**Figure 1:**
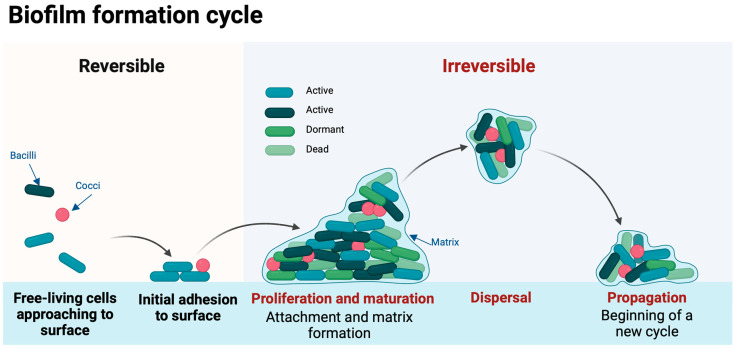
The life cycle of biofilms.

The formation of biofilms encompasses several intricate stages, each crucial in the establishment and resilience of these microbial communities. Initially, there is a reversible attachment phase where macro and micro molecules adhere to surfaces, followed by an irreversible adherence, the proliferation and maturation of the biofilm and last the “seed dispersal” stage, leading to colony formation (Figure 1) [6,7,9]. This attachment occurs on either host tissues or prosthetic materials, marking the initiation of biofilm development [10].

Once firmly attached, bacteria undergo proliferation and secrete molecules to construct a protective matrix, a process known as biofilm maturation [11]. This matrix, comprising proteins, polysaccharides, teichoic acids, and DNA from lysed cells, forms a scaffold that shields the microbial community. Notably, environmental factors like substrate availability and shear forces influence the composition of this matrix [11]. For instance, in coagulase-negative staphylococcal biofilms, polysaccharide poly-N-acetylglucosamine (PNAG) plays a prominent role [12].

Complex signaling and quorum sensing among bacteria facilitate the maturation and stabilization of biofilms. Subsequently, there are discrete episodes of dispersal, where microbes from the biofilm’s outer surface are released, leading to the colonization of nearby or distant surfaces, akin to infection metastasis [13].

Microbes within biofilms exhibit distinct physiological and metabolic characteristics compared to their free-living counterparts. This existence within a biofilm confers numerous survival advantages, notably the resistance to host immune responses and antimicrobial therapies [14]. Biofilm cells demonstrate significantly higher tolerance to antibiotics, ranging from 10 to 1000 times that compared to planktonic cells [15]. This tolerance arises from the metabolic heterogeneity within the biofilm, resulting in differential gene expression patterns and varying metabolic states among cells [16,17]. The diversity observed is linked to varying chemical and nutrient levels across different layers of the biofilm. This variance influences gene expression, leading to cells adopting distinct metabolic states [18]. This heterogeneous growth pattern in biofilms alters the susceptibility to antibiotics by fostering subpopulations with physiological traits that enhance resilience to specific antibiotics [16,17].

Additionally, the biofilm matrix functions as a barrier to antibiotics, reducing their penetration and effectiveness. Antibiotics like vancomycin and cefotaxime, for example, struggle to penetrate staphylococcal biofilm matrices [19,20], which can also trap antibiotics or hinder their action [21,22,23]. The matrix may also serve as a reservoir for enzymes, such as beta-lactamase, that deactivate antibiotics [24]. Furthermore, the horizontal transfer of resistance genes is reported to be 700 times more efficient in biofilms than in planktonic cells [25], significantly increasing the spread of antibiotic resistance [26].

Beyond antibiotic protection, biofilm formation plays a crucial role in evading host immune system attacks. Biofilm cells demonstrate a diminished capacity to activate the innate immune response, leading to reduced inflammation [27]. These cells are recognized for their weakened ability to trigger the innate immune system, resulting in a lower inflammatory reaction.

## 3. Biofilm Infections in Children and in Neonates—Clinical Manifestations and Diagnosis

The formation of biofilms poses a significant health risk to neonates and children, contributing to increased morbidity and mortality. Bacterial biofilms can develop on nearly all medical devices and prostheses (Table 1) [2] and they should be considered in cases of chronic, unresponsive, or recurring infections that are frequently resistant to standard treatments [14].

Common indicators of a biofilm infection include a low-grade fever; mild inflammatory reactions such as redness, pain, and loss of function; and systemic signs of infection that temporarily improve with antibiotic therapy but relapse once the treatment is stopped. Additionally, persistent infections lasting more than a week, failure of antibiotic treatments, recurrent infections with the same pathogen, and signs of antibiotic resistance are key clinical indications. A medical history that includes factors predisposing a patient to biofilm formation should also raise suspicion of biofilm infections [28,29,30].

Laboratory evidence of biofilm infections includes the microscopy of fluid or tissue samples showing microbial aggregates and biofilm structures or aggregates co-localized with inflammatory cells. Positive identification of microbial pathogens through culture-based methods or non-culture techniques, such as polymerase chain reaction (PCR), quantitative PCR, or multiplex PCR, also suggests a biofilm infection. Additional diagnostic methods include fluorescence in situ hybridization (FISH) which shows aggregated microorganisms of known pathogens, and non-culture-based identification techniques such as pyrosequencing and next-generation sequencing. For biofilm infections persisting longer than two weeks, detecting a specific immune response to the identified microorganism can be helpful [28,29,30].

Unlike the well-established diagnosis of infections caused by free-living bacteria, diagnosing biofilm infections has many challenges and lacks consistent reporting [30]. Traditional pathogen identification methods in clinical laboratories, which rely on specific agar and incubation, often fail to detect biofilm-forming microorganisms. These methods, designed for free-living bacteria, miss the biofilms’ unique characteristics [31]. Even with automated systems, microbial identification takes 5–7 days, allowing biofilm-forming pathogens to proliferate, worsening the patient’s condition and often necessitating empirical therapy [32]. This approach risks incorrect antibiotic use and increases patient mortality. Various advanced methods for detecting biofilms are available and are chosen based on the biofilm’s location and the type of sample [30,33,34]. These methods offer greater specificity, sensitivity, and efficiency compared to conventional techniques. A biopsy is considered the most reliable detection method, as it allows for the visualization of microbial aggregates, the extracellular matrix of the biofilm, and immune cells [35]. Larger tissue samples and multiple biopsies enhance the sensitivity and specificity of the results. When biopsies are not feasible, other samples such as sputum, blood, fluids, and secretions can be analyzed [30,36]. However, laboratory analysis of these samples is complicated by the biofilm’s tendency to form microbial aggregates adhered to surfaces [37]. To address this, sonication is employed to dislodge the aggregates from biomaterial surfaces, facilitating further analysis through various methods.

The 2014 guidelines of the European Society for Clinical Microbiology and Infectious Disease recommended detection methods for biofilm laboratory diagnosis such as electron microscopy and PCR. These methods can identify microbial aggregates surrounding inflammatory cells and detect mucoid or small cell variants in positive cultures, which indicate antibiotic resistance [29].

### 3.1. Medical Device-Associated Biofilm Infections

Biofilm formation on medical devices presents significant challenges across various types of devices. Each device type has unique characteristics that influence the composition and behavior of biofilms, as well as the clinical complications they cause.

The epidemiology of biofilm infections in intensive care units has been studied in the literature. Research conducted in two large public hospitals in Brazil revealed that biofilms are present on all frequently touched surfaces in adult, pediatric, and neonatal ICUs, indicating that current cleaning practices are inadequate, while the most common identified organisms, in 51.8% (14 out of 27) of the samples, were *Enterococcus faecium*, *Staphylococcus aureus*, *Klebsiella pneumoniae*, *Acinetobacter baumannii*, *Pseudomonas aeruginosa*, and *Enterobacter* [38]. 

Enteral feeding, a common nutritional practice in NICUs and PICUs, is associated with bacterial contamination of the enteral formula, posing a significant disadvantage. Enteral feeding tubes have been identified as reservoirs for microbial colonization [39,40]. A study by Hurrell et al. found biofilms in 76% of 129 enteral feeding tubes collected from NICUs [39]. The organisms recovered were diverse, primarily including *Staphylococcus* spp., *Enterobacteriaceae* (notably *Enterobacter cancerogenus*, *Serratia marcescens*, *Enterobacter hormaechei*, *Escherichia coli*, and *Klebsiella pneumoniae*), and fungi such as Candida. Many of these isolates carried antibiotic resistance genes [39,40]. Biofilms can lead to clogging of the tubes, compromising nutritional delivery.

Urinary catheters host biofilms with uropathogens such as *Escherichia coli*, *Proteus mirabilis*, *Klebsiella pneumoniae*, and *Enterococcus*. These biofilms can lead to catheter-associated urinary tract infections, which can be difficult to treat and may require catheter replacement [41].

Central venous access devices (CVADs) are among the most commonly implanted devices in children and are crucial for treatment in pediatric oncology and for nutritional support in children with intestinal failure, among other uses [42]. Central venous catheters are affected by biofilms composed of *Staphylococcus epidermidis*, *Staphylococcus aureus*, and *Candida*. They form on both the catheter’s internal lumen and external surface, making them challenging to eradicate without catheter removal [43]. Central line-associated bloodstream infections occur relatively frequently, approximately 0.8 per 1000 line days in general, and up to 3.9 per 1000 line days in hospital settings [44]. These infections are particularly challenging for children who rely on their CVADs for medical care, often necessitating catheter removal to mitigate the risk of relapse, metastatic infection, or persistent bloodstream infection. Peripherally inserted central catheters are also linked to central line-associated bloodstream infections. A systematic review of adult patients examined the comparative risk between peripherally inserted central catheters and central venous catheters. The findings indicated that while peripherally inserted central catheters pose a lower risk of central line-associated bloodstream infections than central venous catheters in outpatient settings, hospitalized patients are equally likely to develop central line-associated bloodstream infections with peripherally inserted central catheters as they are with central venous catheters [45].

Endotracheal tubes develop biofilms containing *Pseudomonas aeruginosa*, *Staphylococcus aureus*, and *Haemophilus influenzae*. Biofilms on endotracheal tubes are particularly challenging to treat due to the protected environment they provide for bacteria [46].

Ventriculoperitoneal shunt infections are another significant issue, with infection rates ranging from 5% to 18% [47], being higher in premature infants or those with post-hemorrhagic hydrocephalus [48]. These shunts are commonly infected with coagulase-negative Staphylococci, *Staphylococcus aureus*, and *Pseudomonas aeruginosa*, all of which are known to form biofilms on prosthetic materials [49]. Such infections are difficult to treat, frequently recurring and often requiring the removal of the shunt and temporary insertion of an extraventricular drain [49]. The biofilms in these devices are particularly concerning due to their proximity to the central nervous system.

### 3.2. Biofilm Formation in Childhood Diseases

Conditions like cystic fibrosis, primary ciliary dyskinesia, and non-cystic fibrosis bronchiectasis compromise the lung’s innate immune function, resulting in the buildup of thick mucus and subsequent infections, primarily infections caused by *Staphylococcus aureus*, followed by *Pseudomonas aeruginosa* [50]. *P. aeruginosa* infections are particularly problematic due to their propensity to form biofilms, which are difficult to eliminate [51,52]. While new drugs targeting the cystic fibrosis transmembrane conductance regulator protein have shown promise in delaying infection onset, they do not offer a complete solution, indicating that biofilm-related infections will likely persist despite the advancements in treatment approaches [53].

### 3.3. Biofilm Formation in Neonates

Neonatal sepsis, a serious condition affecting infants aged 28 days or younger, is characterized by systemic signs indicating infection, along with the presence of a bacterial pathogen in the bloodstream. This condition is stratified based on the timing of symptom onset, delineating early onset sepsis (EOS), which occurs within the first 72 h of life, and late-onset sepsis (LOS), which manifests at 72 h of age or later [54].

Coagulase-negative staphylococci (CoNSs) emerge as the predominant causative agents of LOS in neonates, frequently exhibiting resistance to antibiotic treatment. Their capacity to form biofilms represents a significant virulence factor, posing challenges to infection management. Research spanning a 12-year period has identified a notable portion of *Staphylococcus epidermidis* isolates as biofilm producers, with this trait being associated with antibiotic resistance and an impaired host inflammatory response. Specifically, among 150 neonates experiencing 164 suspected septic episodes with CoNS growth in blood cultures, 61% of *Staphylococcus epidermidis* isolates were biofilm producers, compared to 26% of non-epidermidis CoNS isolates. There was a significant correlation between antibiotic resistance and biofilm production in S. epidermidis, whereas this correlation was not observed in other CoNS species [55]. Moreover, *Staphylococcus capitis*, another opportunistic pathogen prevalent in neonatal bloodstream infections, demonstrated the ability to form biofilms on central venous catheters, reinforcing resistance against antibiotics and host immune defenses [56].

An investigation examining various *Candida* species responsible for bloodstream infections in neonates over a one-year period revealed significant findings. Out of the total isolates analyzed, 41 were identified as producers of biofilms, while the remaining 59 were non-biofilm producers. Among the *Candida* species detected, *C. tropicalis* was the most prevalent, accounting for 43% of the cases, followed by *C. albicans* (41%), *C. krusei* (9%), and *C. parapsilosis* (7%). Notably, there was an observed shift towards a higher prevalence of non-albicans Candida species, which displayed resistance to the commonly used anti-fungal medication fluconazole. Moreover, multi-drug resistance was more frequently encountered among *Candida* isolates that formed biofilms [57]. In a separate case–control study focusing on neonates with a *Candida pelliculosa* infection, certain risk factors were identified. Specifically, the use of three or more broad-spectrum antimicrobials and prolonged hospital stays were associated with an increased likelihood of infection with *C. pelliculosa*. Interestingly, the fungus was not detected on the hands of healthcare workers or in the surrounding environment. Fortunately, all fungal isolates were susceptible to anti-fungal medications, and following appropriate treatment, all infected patients experienced recovery. This underscores the importance of stringent infection prevention and control measures, which effectively mitigated the transmission of infections. The mode of transmission was believed to involve adhesion to the cell surface and subsequent biofilm formation, highlighting the significance of addressing biofilm-related mechanisms in infection control strategies [58]. These findings underscore the critical importance of implementing robust infection prevention and control measures in healthcare settings to curb transmission, as biofilm formation significantly exacerbates the persistence and severity of neonatal infections.

## 4. Biofilm Formation Prevention

Preventive measures targeting biofilm infections in children and neonates underscore strict adherence to infection control protocols, entailing rigorous hygiene practices to combat biofilm formation and transmission. Moreover, judicious use of antimicrobial medications is essential to restrict the emergence of antibiotic-resistant biofilms. Proper cleaning, maintenance, and vigilant monitoring of medical devices are imperative to detect and address any signs of biofilm development effectively. Additionally, minimizing the duration of invasive procedures and medical device usage is crucial to reduce the risk of biofilm-related infections. In NICUs, prudent management involves minimizing the use of materials prone to biofilm formation. Adopting less invasive ventilation techniques, like nCPAP in place of intubation, and promptly removing catheters are two examples of recommended procedures.

To prevent biofilm formation on necessary and irremovable foreign devices, a combination of antimicrobial strategies, proper handling, and rigorous infection control practices is essential. Coating devices with antibiotics, such as minocycline and rifampin, or using silver-coated devices can significantly reduce infection rates and microbial colonization. Antimicrobial polymers also inhibit bacterial adhesion and growth [29,59,60]. Antibiotic lock therapy, which fills the catheter lumen with a high concentration of an antibiotic solution for 12–24 h, is effective for central venous catheters and other long-term devices. Ethanol lock therapy can disrupt biofilms and maintain catheter patency [29,43]. Implementing aseptic techniques during insertion and maintenance, routine flushing with saline or heparin, and regular replacement or maintenance of devices can further prevent biofilm formation [29,61,62]. These measures collectively enhance patient outcomes and the longevity of medical devices.

## 5. Treatment of Biofilm Infections 

### 5.1. Antibiotics

Treating biofilms poses significant hurdles due to their unique structural and behavioral characteristics, rendering them highly tolerant and resistant to antimicrobial therapy. Compared to planktonic bacteria, biofilm-associated bacteria typically require concentrations of antibiotics 10 to 1000 times higher for effective treatment [29]. For instance, when CoNSs are in biofilms, their resistance to antibiotics is increased. Indeed, Qu et al. have shown that the resistance of CoNSs to several antibiotics increases significantly as the biofilm develops [63]. Rifampicin, in combination with penicillin or vancomycin, has demonstrated efficacy against CoNS biofilms, addressing the challenge of resistance often encountered with single-agent therapies [64,65]. 

The mechanisms contributing to the heightened drug resistance of biofilms include slow or incomplete penetration of antimicrobial agents through the biofilm matrix, nutrient depletion in various biofilm layers which slows the growth rate of microorganisms and thereby reduces the number of targets for antimicrobial molecules, and the emergence of bacterial subpopulations in a spore-like, non-dividing state [66,67].

Addressing biofilm infections requires a multifaceted approach, as shown in Table 2. A critical issue of biofilm-based infections is that biofilms are polymicrobial communities in which both bacteria and fungi often occur [67]. Treatment strategies should encompass a wide spectrum of antibiotics with diverse modes of action, and combinations of antibiotics used concurrently or sequentially may enhance efficacy. Early on in the biofilm development process, when the bacteria cells are not yet completely anchored, antibiotics are most effective [6,68,69]. Prolonged antibiotic courses might be necessary to penetrate the biofilm matrix and eradicate bacteria nestled within its structure. Guided by microbial susceptibility testing, antibiotic selection should prioritize optimal efficacy while minimizing the risk of antibiotic resistance development. Vigilant monitoring for a treatment response and adverse effects is essential for the successful management of biofilm infections in pediatric patients.

### 5.2. Foreign Body Removal

Biofilm infections frequently present as long-term illnesses that occasionally deteriorate. While antibiotic therapy can alleviate exacerbations, it cannot fully eradicate bacteria within biofilms [8]. The persistence of biofilms may necessitate more aggressive treatment approaches, including intensified antimicrobial therapy, surgical interventions, or, where applicable, the removal of prosthetic devices [8,14]. Furthermore, a brief course of intravenous antibiotics to eradicate the bacteria that have infiltrated the circulation is necessary.

Addressing biofilm-associated infections can be particularly challenging, especially in children with implant-associated infections where the implanted device is vital for sustaining life, such as the central venous access devices (CVADs), which is used for enteral nutrition in intestinal failure. Due to the complexity and difficulty of removing and replacing implanted devices in infants and young children, salvage attempts with prolonged antibiotic courses are common. However, established guidelines recommend the removal of CVADs infected with specific pathogens like *S. aureus*, *P. aeruginosa*, *Fungi*, or *Mycobacteria* [14,70]. Despite the efforts to salvage infected CVADs, recent trials involving antibiotic-impregnated central venous catheters have failed to reduce infection rates in neonates, highlighting the ongoing need for improved strategies in managing biofilm-associated infections [71].

### 5.3. Novel Approaches

Various innovative strategies are being developed to prevent and treat biofilm infections. These methods are designed to disrupt biofilm attachment, promote biofilm removal, or inhibit biofilm formation by interfering with cellular communication among bacteria through quorum quenching, which entails targeting various stages of biofilm formation [72]. Modifying the chemical or physical properties of biomaterials can prevent the initial attachment of biofilms. For example, the formation of biofilms can be inhibited by the application of antibiotics, biocides, and ion coatings to device surfaces [73]. Furthermore, the hydrophobicity of polymeric materials is altered by hydrophilic polymers, such as hyaluronic acid and poly N-vinylpyrrolidone, which results in a decrease in microbial adhesion [74,75]. Similarly, altering the physical properties of devices, such as using superhydrophobic surfaces, hydrogel coatings, and heparin coatings, can prevent biofilm attachment [76,77]. 

Another area of interest is the development of techniques to eliminate pre-existing biofilms. Matrix-degrading enzymes are specifically engineered to destroy the structures of biofilm matrices, hence enhancing the efficacy of antimicrobial agents [78]. Moreover, certain chemicals such free fatty acids, certain amino acids, and nitric oxide generators have demonstrated the ability to induce biofilm dispersal [79,80]. 

Quorum sensing (QS) plays a pivotal role in biofilm formation, leading to the investigation of QS inhibitors [72]. These compounds disrupt bacterial communication, rendering pathogens more vulnerable to immune responses and antibiotics [81,82]. Strategies include blocking QS molecule biosynthesis, inactivating or degrading QS molecules, and interfering with signal reception. The process of downregulating or silencing the quorum-sensing system is known as quorum quenching. In Gram-negative bacterial pathogens, quorum quenching can be achieved by blocking the biosynthesis of acyl-homoserine lactones (AHL), inactivating or degrading AHL, or interfering with the signal receptor [7]. Scientists have conducted research on chemicals that break down, hinder, or counteract quorum-sensing signals, as well as those that impede signal transduction or transit [72]. These novel approaches have the potential to revolutionize antibiofilm medicines, surpassing the efficacy of existing antibiotic treatments [6,7,8,83].

Moreover, novel electrochemical methods show potential in hindering biofilm formation. By applying a weak electric field in combination with lower doses of antibiotics, known as the “bioelectric effect,” these approaches can disrupt biofilm formation or eliminate mature biofilms, reducing the need for high antibiotic doses [7]. These diverse approaches hold promise for the development of more effective antibiofilm therapies, potentially surpassing the limitations of current antibiotic treatments.

## 6. Conclusions

In conclusion, biofilm infections pose significant challenges in pediatric and neonatal intensive care settings, leading to increased morbidity and mortality rates among vulnerable populations. The complexity of biofilm formation and its resistance to traditional antimicrobial treatments highlight the significance of all-encompassing preventive and therapeutic approaches. Comprising a variety of microbial communities, biofilms flourish on a range of surfaces, but especially on medical equipment. They create robust colonies by means of intricate mechanisms involving attachment, growth, and dispersal. Neonates and children, especially those undergoing intensive care, are at a heightened risk of biofilm-related infections due to their immature immune systems and frequent exposure to invasive procedures and medical devices.

Preventative measures against biofilm infections emphasize strict adherence to infection control protocols, judicious use of antimicrobial agents, regular monitoring, and maintenance of medical devices, along with minimizing the duration of invasive procedures and device usage whenever feasible. In treating biofilm infections, a multifaceted approach is necessary, involving a combination of antibiotics, foreign body removal, and novel therapeutic strategies that target biofilm disruption and inhibition. However, the chronic and recurrent nature of biofilm infections necessitates careful consideration and individualized treatment approaches to achieve successful outcomes.

Emerging research into novel approaches, such as quorum-sensing inhibition, matrix-degrading enzymes, and electrochemical methods, holds promise for more effective antibiofilm therapies in the future. Despite the challenges posed by biofilms, ongoing advancements in understanding their mechanisms and developing targeted interventions offer hope for an improved management of biofilm infections in pediatric and neonatal population.

## Figures and Tables

**Table 1:** Common biofilm infections in neonates and in children.

Common Biofilm Infections in Neonates and in Children	
Device-related infections Endotracheal tubes Central vascular catheters Peripheral vascular catheters Umbilical catheters Urinary catheters Feeding tubes Prosthetic cardiac valves, vascular grafts Orthopedic implants	Tissue infections Chronic otitis media, chronic sinusitis Chronic tonsilitis Dental plaque Endocarditis Lung infection in cystic fibrosis Kidney stones Osteomyelitis

**Table 2:** Combating biofilm infections in children and in neonates.

Methods to Combat Biofilms
Preventive strategies Adherence to infection surveillance protocols Prudent use of antimicrobial agents Regular monitoring and maintenance of medical devices Reducing the length of invasive procedures and device utilization whenever possible Use of less invasive ventilation modalities
Treatment of biofilm-related infections Antimicrobial agents used in combinations, simultaneously, or sequentially Longer duration of antimicrobial therapy Close monitoring of treatment response
Foreign Body Removal
Novel approaches Inhibition of initial biofilm attachment Removal of biofilms Quorum-sensing system blockage
